# Colorimetric Paper-Based Dual Indicator Label for Real-Time Monitoring of Fish Freshness

**DOI:** 10.17113/ftb.60.04.22.7588

**Published:** 2022-12

**Authors:** Bambang Kuswandi, Faridatul Hasanah, Dwi Koko Pratoko, Nia Kristiningrum

**Affiliations:** Chemo and Biosensors Group, Faculty of Pharmacy, University of Jember,; Jl. Kalimantan 37, 68121 Jember, Indonesia

**Keywords:** dual indicators, pH indicator dyes, colorimetric sensor, fish freshness, intelligent packaging

## Abstract

**Research background:**

Fish freshness and quality monitoring are of high importance for consumers, retailers and fishing industry. Therefore, developing novel approaches that are simple, fast, non-destructive and inexpensive to monitor fish freshness in real time is of great value. One alternative is using Intelligent or smart packaging to monitor the freshness or conditions of packaged fish.

**Experimental approach:**

On-package dual indicator label based on paper-based pH sensors was developed for real-time monitoring of the milkfish (*Chanos chanos*) freshness. The paper-based pH sensor was prepared using bromocresol purple (BCP) and bromothymol blue (BTB) that were immobilized onto a filter paper by dip coating. Herein, the fish degradation could be monitored visually by the dual indicator label, where the BCP changes from yellow to pink, then finally to purple, while the BTP changes from orange to green-yellow, and finally to green-blue to indicate fresh, medium fresh or spoiled product, respectively.

**Results and conclusion:**

The label responds to the pH change caused by the fish degradation and the colour of dual indicator changes to show the fish freshness at room temperature and chiller conditions. This pH change was followed by changes in the other parameters related to fish freshness, such as total volatile basic nitrogen (TVBN), total viable count (TVC), texture and odour. The threshold of fish spoilage at room temperature was observed at 8 h and under chiller conditions at 7 days when the deterioration time point was indicated by the colour changes. Thus, it can be concluded that the dual indicator label can be applied as a simple and low-cost on-package active label for fish freshness monitoring.

**Novelty and scientific contribution:**

Increasing consumer concerns about quality and safe food worldwide has boosted the search for a novel approach to food monitoring. In this work, a simple and practical on-package dual indicator label for real-time monitoring of fish freshness was developed. The colorimetric pH sensor was obtained simply by dip-coating of filter paper, yet it enables easy and accurate detection of fish spoilage with the naked eye. Similarly, the dual indicator label changes colour for other freshness parameters, such as TVBN, TVC, texture and odour.

## INTRODUCTION

Fish is a high-value and healthy food that is consumed daily by many people due to its high nutritional value and good taste (*1*). However, storage conditions and treatment after catching the fish can affect strongly its freshness and quality (*2*, *3*). Hence, fish freshness and quality monitoring are of high importance for consumers, retailers and fishing industry. Commonly, the classical methods for the examination of fish freshness are chemical (*4*), microbial techniques (*5*), or sensory evaluation (*6*), which need long analysis time, laborious procedure and skilled operators (*7*, *8*). New and rapid techniques, such as electronic noses (*9*, *10*) and hyperspectral imaging (*11*, *12*) have also been proposed currently. However, these techniques need complicated instruments and are expensive. Thus, developing novel approaches that are simple, fast, non-destructive and inexpensive to monitor fish freshness in real-time is of great value.

Intelligent or smart packaging is a small, low-cost label attached to food packaging to monitor the freshness or conditions of packaged food (*13*, *14*). Commonly, intelligent packaging has an indicator attached to it that allows colour changing by reacting with the chemical compounds released from the packaged food (*15*). For instance, the pH indicator dye (bromocresol green) has been developed as an intelligent label by entrapping the dye in a polymeric membrane to detect fish freshness (*16*). Moreover, other studies based on pH change detection have also been reported recently (*17*-*19*). Herein, a single indicator or sensor was used as an active label for food freshness monitoring. For a more accurate label for food freshness detection, other approaches have been proposed by using mixed pH indicator dyes as an active label for spoilage detection of skinless chicken (*20*) and a dessert (*21*). Even though this label consists of the mixture of two or three pH indicator dyes (*20*), it was still used as a single sensor or indicator having the same drawbacks as traditional acid-base titration with an indicator dye. It is often hard to define when the end of titration is reached, sometimes it is too early or too late. Similarly, with single freshness indicator, the onset of detection associated with the threshold of spoilage is not easy to determine (*22*).

To avoid these disadvantages of a single indicator used, the indicators based on dual pH indicator dyes were developed as a novel approach for the dual sensor label for beef freshness monitoring (*23*). Based on this approach, we have developed a dual indicator label for on-package fish freshness monitoring. The label was made with paper-based pH sensors using bromocresol purple (BCP) and bromothymol blue (BTB). The selected synthetic dyes have several advantages over natural indicators such as the ease and convenience of synthesis, strong colour response, environmental stability, pH sensitivity and a lack of odour. Furthermore, in comparison with a single pH indicator dye, a combination of pH indicators (BCP and BTB) can effectively improve the pH sensitivity to prevent indistinguishable colour transition during the colour change. Paper-based sensors can be made simply by casting, coating or inkjet printing specific reagents, such as pH indicators or complexing agents, onto cellulose-based filter papers (*24*, *25*). They are flexible, sensitive, low-cost and it is easy to make the desired design of freshness label as well as provide the advantage of point-of-need sampling for application in the field, such as in this intelligent packaging, as fish freshness monitoring.

The goal of this work is to construct two pH-sensitive dyes (BCP and BTB) as an on-package dual indicator label for real-time monitoring of fish freshness since both dyes are well known as sensitive indicators in the pH range ≤5.2-≥6.8 (BCP) and ≤6.0-≥7.6, where fish deterioration occurs (*26*). Herein, the dual indicator label response to fish freshness at different temperatures (room and chiller) was correlated with other fish freshness properties, such as pH, TVBN, TVC, texture, and odour, to show the reliability of the label in the real-time fish freshness monitoring.

## MATERIALS AND METHODS

### Chemicals

The dye stock solution of bromocresol purple (BCP) and bromothymol blue (BTB) (Sigma-Aldrich, Merck, Gillingham, UK) was prepared by dissolving 10 mg of BCP or BTP in 10 mL of ethanol (50%) to make 1 mg/mL concentration of each dye. All used chemicals were of analytical grade and used as purchased without further purification.

### Preparation of dual indicator label

**Fig. S1:**
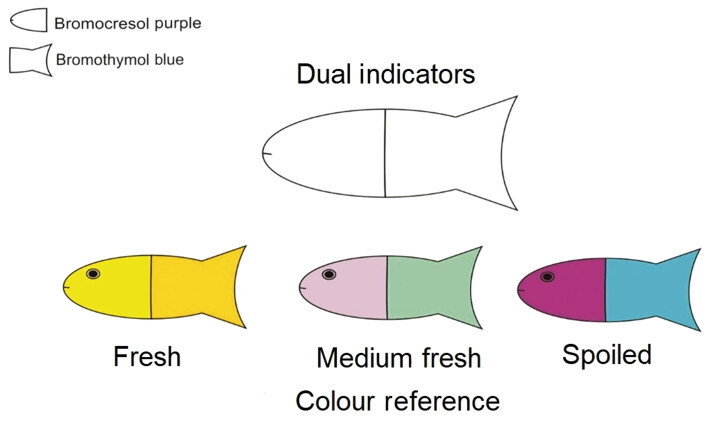
Design of the dual indicator label for fish freshness

The BCP was immobilized on the filter paper (Whatman No. 1001-325; Merck, Burlington, MA, USA) by absorption using a dip-coating technique to create a BCP indicator label by immersing a desired shape into 10 mL of BCP stock solution overnight (12 h) at room temperature. Then, the BCP filter paper was washed with deionized water to remove the unbound dye and completely dried with an electrical dryer. The same dip-coating procedure was used for the immobilization of BTB onto the paper. The paper used for sensor labels was designed in the shape of a fish and placed on the fish package (Fig. S1).

### Preparation of fish samples

Fresh milkfish (*Chanos chanos*) with a pH=6.20 to 6.25 of a similar size and mass (*l*~28 cm, *m*~195 g) was supplied from a local fishpond (Puger, Jember, Indonesia) and packaged using an iced styrofoam box for transporting fish to the laboratory within 25 min. The fish was then cut into two pieces so that it could be placed easily in a styrofoam tray (*I*=20.5 cm, *m*=19 cm, *h*=5 cm). For all experiments similar mass, size and freshness of fish were used. Then, the trays were covered with polyethylene film commonly used for food packaging and stored in the incubator (model MIR 153; Sanyo Electric Co., Osaka, Japan) at room ((28±2) °C) and chiller ((4±0.2) °C) temperatures. The temperature was monitored with electronic temperature device (Cox Tracer®; Cox Technologies Inc, Belmont, NC, USA) during storage. Kinetic analyses of fish samples stored at both temperatures were done at a selected time interval, *i.e.* chemical and microbiological assays, and odour evaluation. For microbiological analysis and odour evaluation, the fish was cut into two portions (~97 g). All measurements were performed in triplicate.

### Characterization of the dual indicator label

Moisture absorption measurements and scanning electron microscopy (SEM) analysis were used for characterization of two indicator labels, plain filter paper used as a substrate, and the filter paper with BCP or BTB dye. The moisture absorption (%) was determined according to a previous method (*27*) with a slight adjustment. The BCP and BTB membranes, and the paper substrate were cut by a hole punch into a round shape (*d*=2 cm). Then, they were placed inside the chamber (Conway diffusion cell), sealed with a cap and stored at 30 °C. The outer chamber was filled with saturated NaCl solution (10 mL). A relative humidity (RH) of 76% was maintained inside the cell. The membrane mass was determined every 12 h until constant, then the moisture absorption (*w*) was calculated as follows:

*w*=[(*m*_t_–*m*_i_)/(*m*_i_)]·100 /1/

where *m*_t_ is the membrane mass (mg) at a certain time interval, and *m*_i_ is the initial membrane mass (mg). Three membrane samples were measured and the moisture adsorption (%) was calculated as an average (the standard deviation).

SEM images of the membrane surface morphologies were obtained at a voltage of 3 kV using an S-4800 SEM (Hitachi, Tokyo, Japan). The samples were prepared for analysis by cutting the membrane into small pieces and sputtering with gold to make the samples conductive. Three samples including plain filter paper, BCP and BTB membranes were examined.

### The dual indicator label response

The dual indicator label was placed inside the package of the fish samples, where it is in contact with the package headspace, while the reference label for reading the freshness degree was placed outside the package, just above the dual indicators. The samples were then stored at room and chiller temperatures to evaluate the performance of the labels for fish spoilage monitoring.

Since the dual indicator label could be read by the naked eye, the colour changes of the label were taken by scanometric method (*28*, *29*) using a flatbed scanner (Canoscan, LIDE 110, Tokyo, Japan), with the colour image resolution set at 300 dots-per-inch (dpi). Then the ImageJ® program for Windows® (*30*) was employed to analyze the colour value. The label colour response was presented as a mean RGB value. All of the measurements were done in triplicate.

### pH, volatile amine and microbiological analysis

The fish sample pH values were measured in triplicate by a pH meter (model RL060P; Russell, Richmond, VA, USA), where the glass electrode was immersed in the homogenate fish sample solution. The total volatile basic nitrogen (TVBN) values were obtained and analyzed using digestion of fish samples with perchloric acid (PCA) according to Pearson (*31*). All the fish samples were thoroughly washed with tap water. Then, the fish were aseptically skinned and minced by passing three times through a grinder (4 mm holes). The fish samples (10 g) were blended with 90 mL of PCA (6%) and then the filtrate (50 mL) was alkalinized with 0.1 M sodium hydroxide (*20*) and distilled in a Kjeltec^TM^ 2100 distillation unit (FOSS Analytical, Hillerød, Denmark) for 10 min (*32*). The TVBN analysis was performed in duplicate.

The microbiological analysis was done according to Kuswandi *et al.* (*32*) with slight modification. The fish samples (25 g) were weighed aseptically, then added to strength Ringer's solution (225 mL) and homogenized in a stomacher (Lab Blender 400; Seward Medical, London, UK) for 1 h at room temperature. Serial dilutions in Ringer's solution (¼ strength) were made and duplicate samples (1 mL) of proper dilutions were spread on the surface of the medium in Petri dishes for enumeration of total viable count (TVC) of aerobic bacteria using plate count agar (Merck, Darmstadt, Germany) and incubated at 25 °C for 72 h. The plate was visually inspected for the typical colony and morphological types that were associated with the growth medium. Colonies were calculated and presented as log CFU/g.

### Odour and texture

The sensory evaluation of the fish sample was done at chiller or room temperatures by 20-member trained panel consisting of students from the department (10 males and 10 females between 19 and 21 years old). The panellists were trained to select their preferences objectively according to the odour acceptance of the fresh fish samples. Odour was evaluated with descriptive terms that reflected the organoleptic change of quality degradation (*33*). The odour training session used five fish samples at different stages of freshness (fresh, medium fresh and spoiled). The trained panellists were not informed about the status of each fish sample regarding storage time and temperature. The odour was evaluated under ambient conditions, *e.g.*ventilation and lighting, where the light and temperature of packaged fish samples ware stored similar to real condition in the fish market. The freshness of fish samples was determined by evaluating its odour with score 1 for like and 0 for dislike (*34*). In other words, if all panellists evaluate the fish odour as acceptable, the sample is fresh (100%), but if all panellists dislike the fish odour, the sample is spoiled (0%). The medium status of odour acceptance is when 50% or more panellists evaluate samples as acceptable. The fish texture was measured with a texture meter (TA-XT2i; Stable Micro Systems, Godalming, UK), which was used to compress fish meat between two parallel plates at a crosshead speed of 1 mm/s to give a more objective evaluation.

### Statistical analysis

The statistical analysis of data (mean value±standard deviation) for moisture absorption properties of plain filter paper (control), and the paper with immobilized BCP and BTB was done using SPSS v. 16.0 (*35*). An analysis of variance (one-way ANOVA) using the linear model procedure was performed. Descriptive and Tukey's multiple range tests were conducted for comparing the mean values at a 5% significance level.

## RESULTS AND DISCUSSION

### Colorimetric determination of fish freshness

Filter paper is a common substrate for making indicator labels because it is readily available at low cost. Here, the sensing membrane was obtained by dip-coating of filter paper with pH-sensitive dye *via* simple adsorption as described above. The label was designed in the shape of a fish (Fig. S1), since it is simple in preparation and design (by cutting with scissors or a cutter into a desired shape) and the freshness level can be easily distinguished by the naked eye (*23*). Both parts (head and tail) can be monitored with the BCP and BTP membrane, respectively. Finally, both dry pH membranes could be constructed to produce a paper-based pH sensor with the aid of transparent double tape, with one side forming the dual indicator label, and the other attached inside fish packaging. For simple colorimetric detection of different stages of fish freshness (*i.e.* fresh, medium fresh and spoiled), the label can be read easily by the naked eye. Furthermore, this label construction prevents the two indicator dyes from diffusing across each other inside the filter paper, which in turn, could change the label colour. Both dry BCP and BTB membranes were separated using plain filter paper, and transparent double tapes were used only to hold the membranes in their position.

**Fig. 1:**
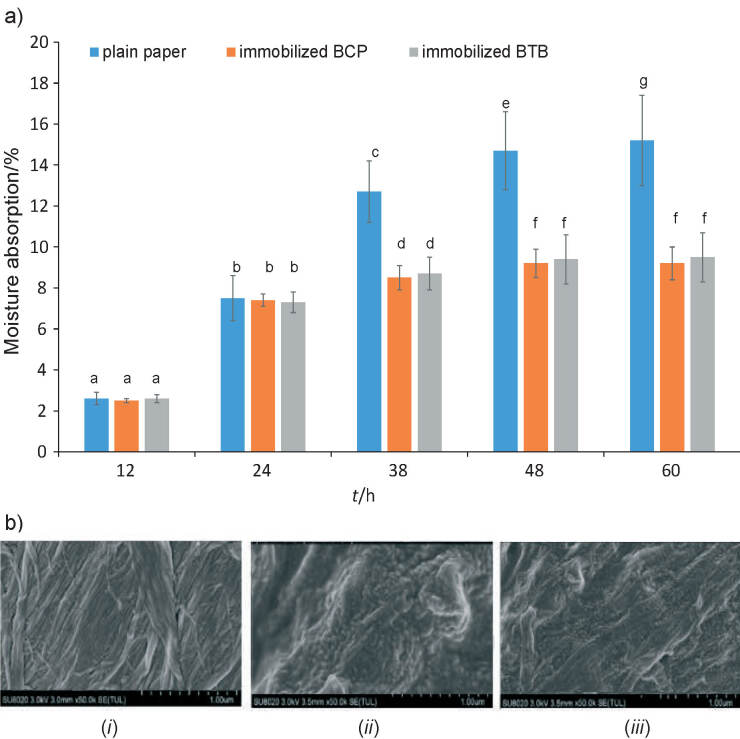
Moisture absorption properties of plain filter paper (control), and paper with immobilized bromocresol purple (BCP) or bromothymol blue (BTB): a) the bars with the same letters indicate not significant difference (p˂0.05), and b) scanning electron microscope images of: (*i*) plain filter paper, and the paper with: (*ii*) immobilized BCP and (*iii*) BTB

Moisture absorption is one of the important characteristics of a colorimetric indicator (*36*). When a colorimetric indicator is attached to the packaging headspace, high relative humidity (RH) in the package might have a negative effect on its colour change, which can lead to an false response because of its swelling or damage after moisture absorption. Therefore, low moisture absorption by the indicator is allowed. Here, the moisture absorption of all three samples increased and reached saturation over time. The moisture absorption at 60 h and 76% RH by the filter paper, BCP and BTB membranes was 15.2, 9.2 and 9.5%, respectively (Fig. 1a). It was found that the moisture absorption of the indicator membranes (BCP and BTB) was almost twice as low as of the plain filter paper (p˂0.05). This finding of the moisture content of the indicator membrane (>100%) is enough to reduce moisture from interfering with the indicator response in terms of its colour change (*36*, *37*).

The SEM images of three samples (filter paper, BCP and BTB membranes) under magnification of 50 000 show clear differences among them (Fig. 1b). Therefore, it can be stated that the indicator solution was evenly adsorbed to the surface of filter paper so that it could bind BCP and BTB dye molecules tightly with good stability (*38*). Moreover, fibrous and fluffy surfaces also appeared on the BCP and BTB membranes, so that they served as a larger surface area to interact instantaneously with the released gas from the fish sample (*39*). Thus, the surface properties of both indicator membranes are suitable for an active label application.

### Response of dual indicator label to the pH change

**Fig. 2:**
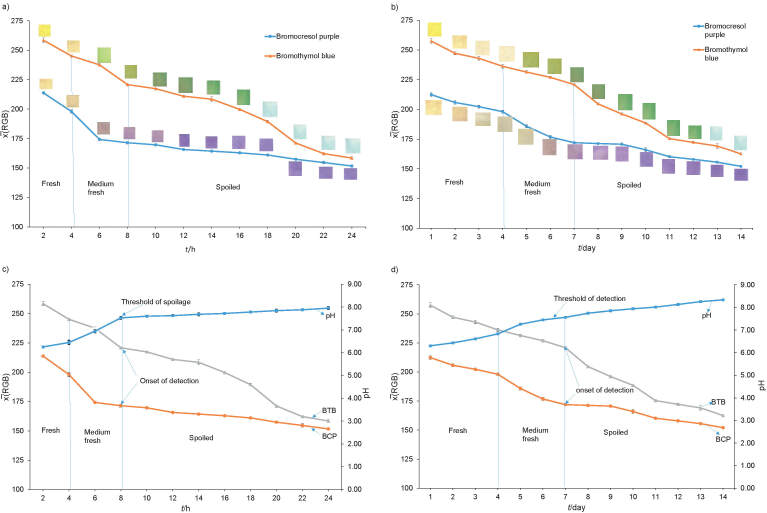
The rate of the dual indicator label change as the colour response (mean RGB) towards fish spoilage at: a) room and b) chiller temperatures, and the pH values of packaged fish samples and the dual indicator label responses at: c) room and d) chiller temperatures. BTB=bromothymol blue, BCP=bromocresol purple

All dual indicator labels were attached inside the headspace packaging close to the fish samples to enable the interaction with the volatile amines produced by the degrading fish samples. The very distinctive colour change of the BCP membrane (head indicator) from yellow to pink and finally to purple, and that of the BTP membrane (tail) from orange to green-yellow and finally to green-blue indicated fresh, medium fresh and spoiled fish, respectively (*23*, *40*). The label was inspected periodically until no further colour development was detected. The rate of the colour changes of the dual indicator labels as a response to fish spoilage at room and chiller temperatures is expressed as mean RGB values (Figs. 2a and 2b). Here, both the BCP and BTB membrane responses decreased sharply up to 8 h, when they changed slightly up to 24 h of the experiment, where BCP was almost steady after the colour changed to purple at room temperature (Fig. 2a). At the chiller temperature, both BCP and BTB membranes changed colour sharply during the first 4 days, then gradually up to day 14, when BCP was almost stable after the colour changed to purple (Fig. 2b). Moreover, the visual detection by the naked eye did not find any variation in the colour development among these labels of different batches under both conditions. Thus, the fish samples had the same freshness that roughly produced the same mass fraction of volatile amines, which in turn, caused roughly the same pH change inside the headspace of the package, resulting in the same colour change of the dual indicator label. The onset of fish spoilage was found after 8 h and 7 days at room and chiller temperatures, respectively (*39*, *41*). The results also show that volatile amines were produced gradually from the fish samples during the deterioration process.

Here, by developing the dual indicator label as a real-time active label, the false-negative and false-positive response could be prevented, as both indicator membranes show similar pattern in colour change, thus disabling false or erroneous response. Placing them inside the package enables their direct contact with the headspace for fish freshness monitoring and avoids the interference from the environment (*23*). The false negative result might happen if the plastic cover was opened or broken. The volatile amines released during fish sample degradation would evaporate, causing their concentration inside the headspace to decrease significantly, which would affect the response of the label. Hence, keeping the package intact during storage and the label in its position is compulsory for reliable monitoring of fish freshness (*23*).

The precision of the dual indicator label response to fish freshness is shown with the reproducibility of the colour development as the error bars (Figs. 2a and 2b), where their values were below 2%, which is excellent for this type of measurement (*42*). Furthermore, the robustness of the label was evaluated in different batches and at different storage days to evaluate their response to fish freshness (*42*). The results showed that their responses were consistent with different fish freshness.

The pH value of the packaged fish samples along with the dual indicator label responses at room and chiller temperature are shown in Figs. 2c and 2d. It increased steadily from pH=6.20 when fresh to pH=7.53 at the threshold of spoilage at 8 h, while the dual indicators also develop their colour as the onset of detection at room temperature (Fig. 2c). At chiller temperature, the pH value of the fish samples increased steadily from pH=6.25 on day 1 to the spoilage threshold pH=7.56 on day 7, which corresponded to the colour change of the dual indicator label (Fig. 2d) (*43*, *44*). According to Fig. 2, both indicator membranes also show decreased mean RGB value as the pH increased at both temperatures. This is because the colour of the both BCP and BTB pH sensitive indicators changes at the similar pH range as the fish sample freshness. Generally, spoilage of fish occurs at pH>7.0, while pH of fresh fish is <6.0 (*2*, *4*). This pH value was achieved at 8 h and 7 days at room and chiller temperature, respectively (*39*, *41*). Hence, the labels show a correct response to the onset of spoilage (when fish spoilage just started) after 8 h and 7 days at the same temperatures.

### TVBN and microbial analyses of fish samples

Commonly in spoiled fish, the total volatile basic nitrogen (TVBN) value increases because of the NH_3_ production, including other volatile amines, as a result of protein degradation. Moreover, fish deterioration can be detected by the production of biogenic amines (*e.g*. histamine, tryptamine, tyramine, spermidine, spermine, cadaverine and putrescine), produced *post mortem* in the fish and shellfish products (*45*). These biogenic amines are low-molecular-mass aliphatic, alicyclic or heterocyclic organic bases that originate from the specific free amino acid decarboxylation in fish or shellfish tissue (*3*, *46*). The use of TVBN as objective product standards or the indices of fish quality has long been used and suggested, due to the tests being rapid if compared to microbiological analyses and less subject to individual interpretation than sensory analyses (*47*).

**Fig. 3:**
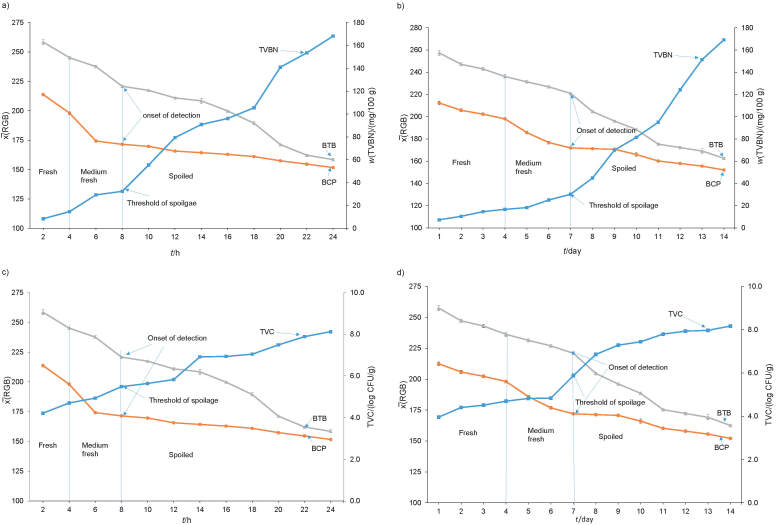
The total volatile basic nitrogen (TVBN) values of packaged fish samples and the dual indicator label responses at: a) room and b) chiller temperature, and the total viable count (TVC) value of packaged fish samples and the dual indicator label response at: c) room and d) chiller temperature. BTB=bromothymol blue, BCP=bromocresol purple

Figs. 3a and 3b show that the TVBN mass fraction increased from 7.355 in a fresh sample to 32.376 mg/100 g as a threshold of spoilage at 8 h at room temperature, and from 7.311 to 30.288 mg/100 g at 7 days at chiller temperature, as the onset of detection when dual indicators start to change the colour. Hence, the dual indicators properly respond to the TVBN increase in the fish package headspace, as the pH range of dual indicator colour development is associated with the TVBN value in the fish sample. Here, the fish freshness decreased when TVBN increased, where a TVBN value of fresh fish is limited to <35 mg/100 g for many various species (*48*). The TVBN values were detected at 8 h and 7 days as the onset of spoilage detection, where the colour of the dual indicator label changed to show that the packaged fish was spoiled under both conditions (Figs. 3a and 3b) (*32*). Thus, it can be stated that the dual indicator label can be employed as a simple and effective active label to indicate the high TVBN value in packaged fish with its colour change, which is observable by the naked eye.

The microbial analysis of the fish sample was presented as the total viable count (TVC) (Figs. 3c and 3d). According to the results, during the first 2 h, the TVC steadily increased from 4.213 to 5.487 log CFU/g, which presents a threshold of fish spoilage at room temperature. This point is also an onset of detection of the dual indicator colour change (Fig. 3c). At the chiller temperature, the TVC increased from 3.960 to 5.875 log CFU/g on day 7 as a threshold of fish spoilage, and as the onset of detection of the dual colour change (Fig. 3d). According to Figs. 3c and 3d, the decrease of mean RGB values as a measure of dual indicator colour response is proportional to the increase of TVC. The threshold value of TVC≤7.0 log CFU/g was used for detection of bacterial spoilage of fish in many studies (*12*). Here, the TVC value used to determine the spoiled fish was obtained slightly earlier, *i.e.* at 8 h and 7 days at room and chiller temperature, respectively. Hence, this value prevents false-positive label response in the microbial growth detection, compared to previously used on-package labels with BCP as a single indicator (*27*). The dual indicator label gave a reliable response to the increase of the bacterial population in the fish sample at both temperatures, which makes it a simple and effective tool for the rough estimation of microbial count and detection of fish spoilage.

### Texture and odour analysis of fish samples

**Fig. 4:**
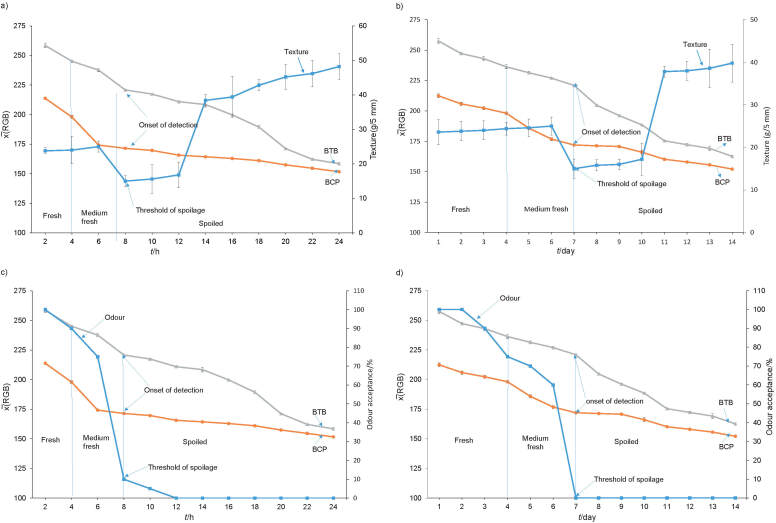
The texture values of packaged fish samples and the dual indicator label responses at: a) room and b) chiller temperature. Odour (%) of packaged fish samples and the dual indicator label responses at: c) room and d) chiller temperature. BTB=bromothymol blue, BCP=bromocresol purple

The average softness of fish samples at room and chiller temperature in triplicate measurements are shown in Figs. 4a and 4b, respectively. The freshness deteriorates with the increase of the softness of fish samples (*49*). It was concluded that the dual indicator label can be employed to indicate the increase in the softness of fish samples, as this value indicates their deterioration.

**Fig. S2:**
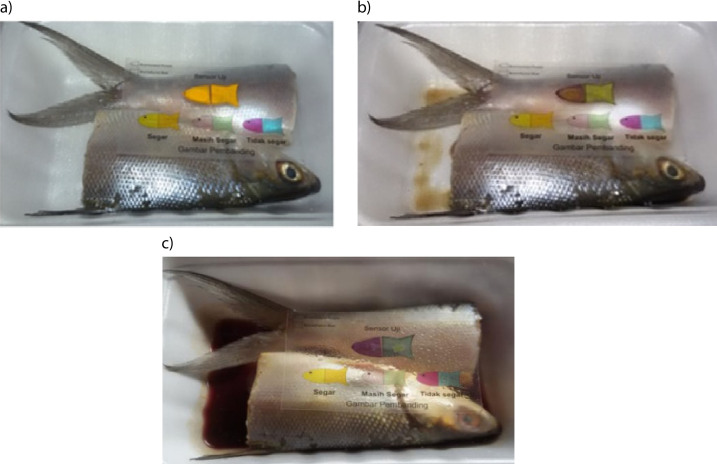
The dual indicator label responses to a different level of fish freshness: a) fresh, b) medium fresh and c) spoiled

As fish odour is an important sensory property, it needed to be evaluated parallel with the dual indicator label response to fish freshness. The odour of fish samples was evaluated in the laboratory, without regulating the conditions of the analysis, as if the label was applied at home or in the shops. The odour acceptance or odour score (%) of the fish samples evaluated at room and chiller temperature is presented in Figs. 4c and 4d respectively. It is shown that the dual indicator label response is similar to declined value of odour acceptance, caused by increasing microbiological activity inside fish (*2*), where the point of rejection of odour is 10% at room and 0% at the chiller temperature according to our setting for odour rejection, which was associated with the onset of detection of the dual indicator label colour change (Figs. 4c and 4d) (*36*, *49*). The on-package dual indicator label was successfully applied on the packaged samples as a reliable active label for monitoring fish freshness at three stages: fresh, medium fresh and spoiled (Fig. S2).

## CONCLUSIONS

An on-package dual indicator label based on colorimetric paper-based pH sensors using bromocresol purple (BCP) and bromothymol blue (BTB) was developed for monitoring fish freshness. According to the results, the dual indicator label can be employed for determining fish freshness. The correlation between the dual indicator label colour change and the fish degradation over time represents the fish spoilage status visually (when the BCP membrane changes to purple and BTB membrane to green-blue). The dual indicator label reacts properly to the changes in fish freshness as its colour change corresponds to the deterioration time points (fresh, medium fresh and spoiled). This is due to the improvement of moisture resistance of the sensing label by simple adsorption of the reagent dyes, which prevents moisture from interfering with the accuracy of the dual indicator label. In conclusion, the label can be feasibly employed as a simple, real-time active device for effective monitoring of fish freshness that can be used for optimizing distribution and control of the fish product sales rotation system, *i.e.* which fish product should be sold in a few hours, thus reducing fish waste and loss.
